# Supplementary material for: Combinations of maternal-specific repressive epigenetic marks in the endosperm control seed dormancy
Source: eLife. 2021 Aug 24;10:e64593. doi: 10.7554/eLife.64593 (PMC8456740; doi:10.7554/eLife.64593)
Supplement: Supplementary file 1. [file elife-64593-supp1.docx]

| Target gene | Locus ID | Oligonucleotide name | Sequence (5´to 3´) |
| --- | --- | --- | --- |
| Primers for vector constructions | | | |
| *EPR1* promoter | AT1G18330 | EPR1pro_f_KpnI | TAGGTACCCTTCACGACATCCTCCATATCTC |
|  |  | EPR1pro_r_XbaI | CGTCTAGACTTGTAAGTGATTTACTGAGAAC |
| *TPS1* promoter | AT1G78580 | TPS1pro_f_KpnI | TAGGTACCCTCAAGCCAACTCTGCTTCTATC |
|  |  | TPS1pro_r_XbaI | GCTCTAGAACGCTCACACCAAAACAGAAC |
| *REF6* | AT3G48430 | REF6_f_AatII_infusion | TCTAGAGGGCCCGACGTATGGCGGTTTCAGAGCAGAGTCAAG |
|  |  | REF6_r_Eco32I_infusion | GGCCGCACTAGTGATTCACCTTTTGTTGGTCTTCTTAACC |
| Primers for qRT-PCR | | | |
| *EDF4* | AT1G13260 | EDF4_rt_f | AAAGACGTGAAGATGGACGAAGA |
|  |  | EDF4_rt_r | GCCGTTTACTCTGCTCTAACTCT |
| *ABI3* | AT3G24650 | ABI3_rt_f | TTAAAGTAAGACAACCGAGCGGA |
|  |  | ABI3_rt_r | TGTTCCTTTGCGACTTGTTTTGT |
| *HSFC1* | AT3G24520 | HSFC1_rt_f | ACAAAAAGCAAGCCACGTCAATA |
|  |  | HSFC1_rt_r | AAATTAAAGAGGAGCCTGGAGGG |
| *REF6* | AT3G48430 | REF6_rt_f | GCAGACGTTTTCACTTTCAGACA |
|  |  | REF6_rt_r | ACAATGCTTTTCCTACACCAGGA |
| Primers for genotyping PCR | | | |
| *REF6* | AT3G48430 | ref6-1_genotype_LP | TCATATACAAGGCGTTCGGTC |
|  |  | ref6-1_genotype_RP | CAGTTGCAACTCTGGAGAAGG |
| *REF6* | AT3G48430 | ref6-3_genotype_LP | AGGTTTGGATGTCACATCAGG |
|  |  | ref6-3_genotype_RP | CAGTTGCAACTCTGGAGAAGG |
| *ELF6* | AT5G04240 | elf6-3_genotype_LP | ACGTCAATGCGGTAATCATTC |
|  |  | elf6-3_genotype_RP | TTTGCAGATCCCATTGCTTAC |
| *ELF6* | AT5G04240 | elf6-4_genotype_LP | TGACGGACCAAGAAAATTGTC |
|  |  | elf6-4_genotype_RP | ATTCCAAAGAGAGGCCATTTC |
| - | - | SALK_LB | ATTTTGCCGATTTCGGAAC |
| - | - | Sail_LB | GCCTTTTCAGAAATGGATAAATAGCCTTGCTTCC |
